# Fluorescence-guided resection of tumors in mouse models of oral cancer

**DOI:** 10.1038/s41598-020-67958-8

**Published:** 2020-07-07

**Authors:** Paula Demétrio de Souza França, Navjot Guru, Sheryl Roberts, Susanne Kossatz, Christian Mason, Marcio Abrahão, Ronald A. Ghossein, Snehal G. Patel, Thomas Reiner

**Affiliations:** 1 Department of Radiology, Memorial Sloan Kettering Cancer Center, 1275 York Avenue, New York, NY 10065 USA; 2 Department of Otorhinolaryngology and Head and Neck Surgery, Federal University of São Paulo, São Paulo, SP Brazil; 3 Department of Nuclear Medicine, School of Medicine, Technische Universität München, Munich, Germany; 4 Department of Pathology, Memorial Sloan Kettering Cancer Center, New York, NY USA; 5 Department of Surgery, Memorial Sloan Kettering Cancer Center, New York, NY USA; 6 Department of Otorhinolaryngology, Weill Cornell Medical College, New York, NY USA; 7 Chemical Biology Program, Memorial Sloan Kettering Cancer Center, New York, NY USA

**Keywords:** Surgical oncology, Single-strand DNA breaks

## Abstract

Complete removal and negative margins are the goal of any surgical resection of primary oral cavity carcinoma. Current approaches to determine tumor boundaries rely heavily on surgeons’ expertise, and final histopathological reports are usually only available days after surgery, precluding contemporaneous re-assessment of positive margins. Intraoperative optical imaging could address this unmet clinical need. Using mouse models of oral cavity carcinoma, we demonstrated that PARPi-FL, a fluorescent PARP inhibitor targeting the enzyme PARP1/2, can delineate oral cancer and accurately identify positive margins, both macroscopically and at cellular resolution. PARPi-FL also allowed identification of compromised margins based on fluorescence hotspots, which were not seen in margin-negative resections and control tongues. PARPi-FL was further able to differentiate tumor from low-grade dysplasia. Intravenous injection of PARPi-FL has significant potential for clinical translation and could aid surgeons in assessing oral cancer margins in vivo.

## Introduction

An estimated 53,000 people were diagnosed with oral and oropharyngeal cancer in the US in 2019^1^. Squamous cell carcinoma (SCC) is by far the most common epithelial malignancy in the oral cavity, accounting for over 90% of all cases^2,3^. This cancer is mostly related to lifestyle, with the major risk factors—tobacco and alcohol misuse—increasing the risk of oral SCC (OSCC) up to 25-fold^4^. Although risk factors for OSCC are well-established, and despite the markedly decreased use of tobacco products, the disease’s incidence and mortality continue to increase in the US. Other risk factors include human papilloma virus (HPV) infection, which is associated with an increase of SCC in oropharyngeal cancer^5^. Nevertheless, HPV infections do not explain the increased incidence of tongue OSCC in young women^5^.

Surgical resection of the primary tumor with negative margins is the gold standard treatment for OSCC^6,7^. It is widely known that negative margins in surgical resection are strongly associated with a lower risk of local recurrence and higher survival rates^7–9^. Classically, the gold standard for negative-margin resection is the histological presence of normal tissue surrounding the tumor to a distance of at least 5 mm^6^. This usually requires the arbitrary removal of large amounts of healthy tissue, often leads to large surgical defects requiring complex procedures for reconstruction and, depending on the location of the tumor, may cause irreversible impairment of phonation, mastication, gustation, and swallowing^9^. Our group has proposed to redefine close surgical margins in oral cavity squamous cell carcinoma. In our data local recurrence-free survival was significantly affected only with surgical margins of less than 2.2 mm. This novel definition of close margins stratifies patients for local recurrence better than the arbitrary 5 mm cutoff that has been used for decades^6^.

Despite this new cutoff in OSCC, if histologic margins are smaller than 5 mm, administration of adjuvant treatment remains, in most cases, the standard of care^10,11^. Although widely used in clinical practice to determine the administration of adjuvant treatment, the measurement of the width between the edge of resection and the tumor is subject to various influences, including imprecise measurements^6^. Besides this, it is important to consider that the final histopathological report on margin status typically becomes available only days after surgery, by which time reassessment of the surgical defect for additional complementary resection is much more complicated, and sometimes even impossible. A quicker alternative to this would be intraoperative frozen tissue sections, with results usually available within 15 to 20 min of sampling^12^. However, the negative predictive value of this technique is poor, meaning that negative margins on frozen sections do not guarantee margin-negative resections^13^, mostly due to tissue sampling errors. With more specific methods being unavailable, surgeons rely heavily on imprecise visual inspection and palpation^14^.

Intuitively, a technology that would improve intraoperative margin assessment is an unmet clinical need. Here, we explore the use of an intravenously injected fluorescent imaging agent, PARPi-FL, as a potential tool to help surgeons identify positive margins in real time. PARPi-FL targets PARP1/2, and its use as an imaging tracer, both preclinically^15–17^ and clinically^18^, is well established.

PARPi-FL crosses the cellular membrane, the nuclear membrane and binds to PARP1/2. PARPi-FL uptake specificity was previously reported by correlation of PARP1 protein expression and PARPi-FL retention, as well as by the ability to block the uptake by saturating the enzyme with olaparib or other PARP inhibitors^16,19–23^. Other imaging modalities, including radiolabeled PARP inhibitors^24^, have been explored as well, and some have been translated to the clinic (see^19^ for an overview).

Here, we describe the use of PARPi-FL in mouse models of oral cancer, including both orthotopic xenografts and a chemically induced mouse model. We confirm that PARP1 expression is higher in cancer cells and lower in normal and dysplastic oral tissues^18,25,26^, making it possible to differentiate between them. Using two clinical imaging systems, both for macroscopic and cellular imaging, we were able to delineate oral cancer and accurately identify positive margins in mice. Intraoperative visualization of PARP1 could potentially lead to sensitive and specific imaging of oral cancer, helping clinicians to assess surgical margins in real time.

## Results

### PARP1 is overexpressed in mouse models of tongue cancer

**Figure 1:**
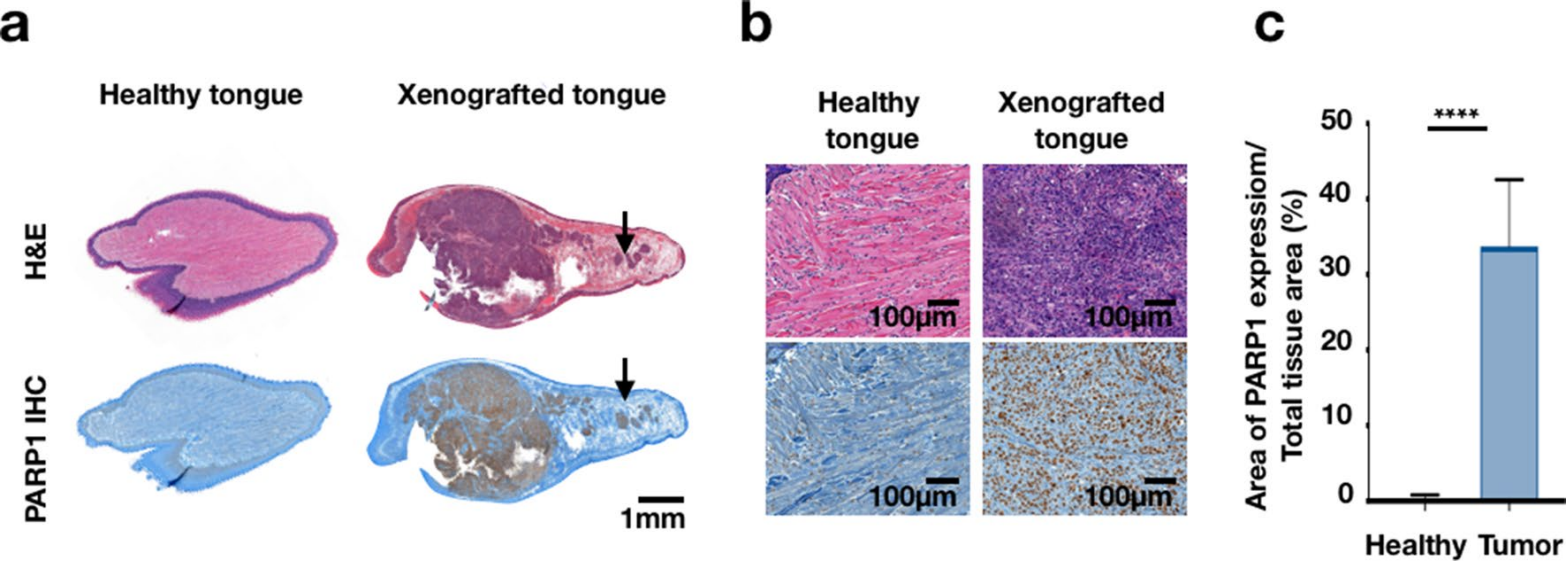
PARP1 expression allows tumor delineation. (**a**) Sagittal cut of a tongue from a normal and a xenografted mouse, H&E (top) and PARP1 IHC (bottom). Histologically, squamous cell carcinoma is known to present small tumor islands (arrows) next to the primary tumor. (**b**) Healthy and xenografted tongue represented in lower and higher magnification. Very low PARP1 expression was seen in normal muscle, whereas abundant PARP1 expression was seen in tumor. (**c**) Quantification of PARP1 expression in tumor compared to normal muscle (*p* < 0.0001).

With an orthotopic mouse model, we observed the presence of microscopic tumor islands around the main tumor site, an archetypical feature of squamous cell carcinoma (Fig. 1a). Having H&E as gold standard and using immunohistochemistry for quantification, we demonstrated that the overexpression of PARP1 is distinctly higher in these tumor masses compared to the normal tissue around them (Fig. 1b). PARP1 expression in the normal tongue was significantly lower, 0.37 ± 0.45% of PARP-positive area over the total tissue area, when compared to tumor, 33.73 ± 8.84% of PARP-positive area over the total tissue area (*P* < 0.001, Fig. 1c).

### PARPi-FL accumulates in tumor and in metastatic lymph nodes

Whole-body sectioning and imaging were performed in an orthotopically xenografted mouse model of tongue cancer; we observed stronger accumulation of PARPi-FL in the tumor and no signal in the surrounding muscle tissue of the tongue (Supplementary Fig. 1. PARPi-FL also accumulated in the neck metastatic lymph nodes but not in the normal structures. Autofluorescence from the skin, whiskers, nose turbinate, and from the normal tongue’s keratinized epithelium was also detected and appeared in the green fluorescence channel. These autofluorescence signals were seen in both the tumor-bearing and control mouse, while tumor and lymph node signals were only detected in the tumor-bearing mouse. In addition, autofluorescence from the food, present in the animal’s digestive system, was also detected (Supplementary Fig. 2).

### PARPi-FL can identify compromised margins

**Figure 2:**
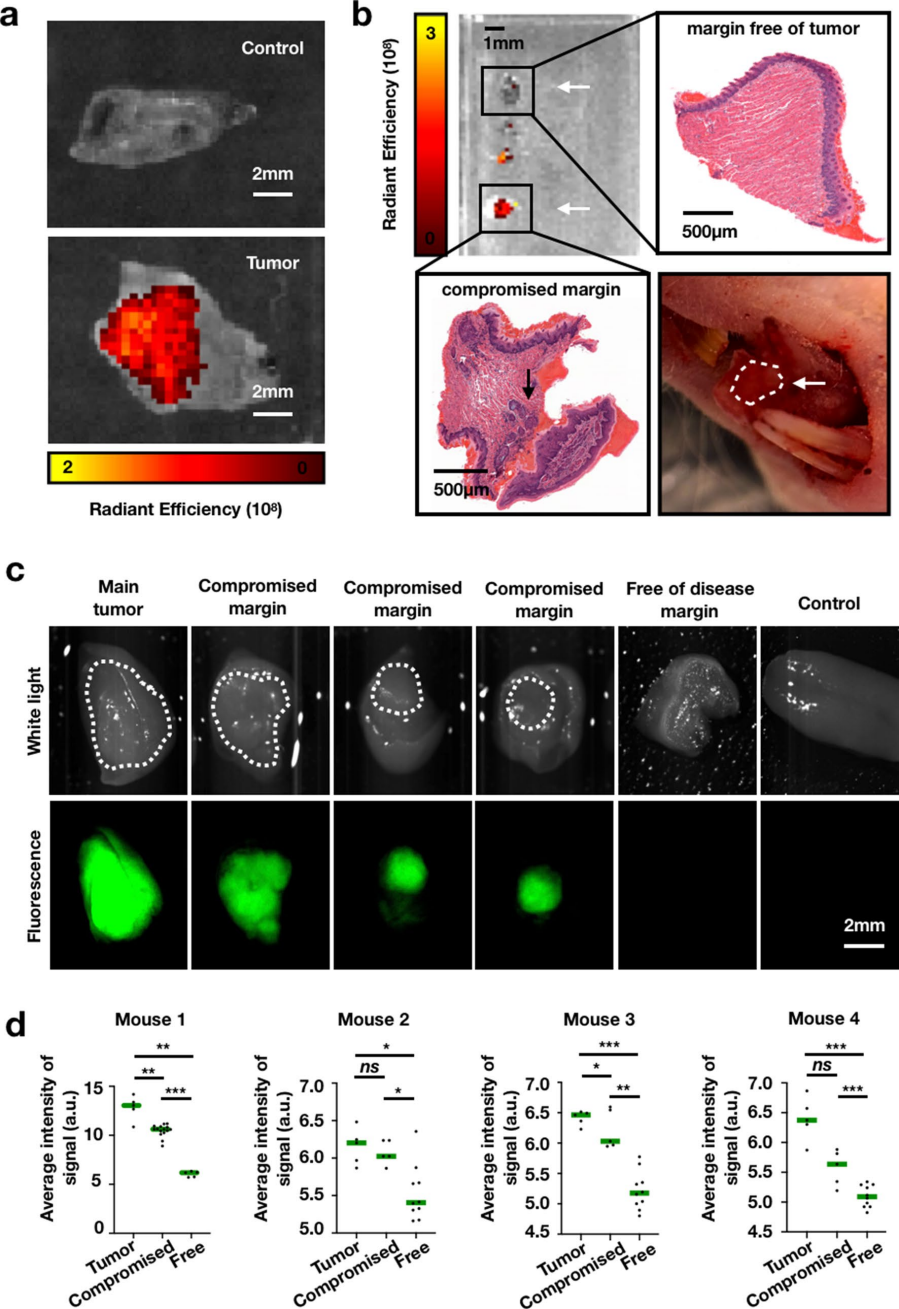
Imaging of orthotopically tumor-bearing mice after intravenous administration of PARPi-FL. (**a**) IVIS imaging of tumor delineation (GFP Filterset—excitation: 465/30 nm, emission: 520–580 nm) with subsequent removal of autofluorescence through spectral unmixing. Representative imaging of a control and orthotopic mouse intravenously injected with PARPi-FL. PARPI-FL accumulates in the tumor but not in the normal muscle around it, delineating the lesion. (**b**) Correlation of PARPi-FL accumulation with H&E histology showed that PARPi-FL signal was only present in tumor-bearing tongues and positive margins. (**c**) Stereoscope imaging of margins (excitation: 470/40 nm, emission: 525–50 nm). As with the IVIS, PARPi-FL signal could be detected in the compromised margins but not in the disease-free tissue. Dotted circles in the white light represent the tumor area. (**d**) Quantification of the average signal intensity, discriminating tumor, compromised, and negative margins in all animals.

In order to take advantage of PARP1 overexpression in tumors, we intravenously injected PARPi-FL and imaged the tumors using both a preclinical IVIS epifluorescence imaging system and a Lumar stereoscope. With the IVIS imaging system, we identified PARPi-FL in areas of the tongue where tumors were present, whereas lower signal was observed in areas with normal muscle and in the control tongues (Fig. 2a). The average epifluorescence signal in the tumor areas within the xenografted tongues was 4.98 × 10^7^ ± 4.48 × 10^7^ (5.59 × 10^7^ ± 5.69 × 10^7^ for FaDu and 4.08 × 10^7^ ± 2.10 × 10^7^ for Cal27) average radiance efficiency, whereas in normal tongue the signal was 0.65 × 10^7^ ± 0.26 × 10^7^), yielding a tumor-to-background ratio of 7.6 (10.3 for FaDu and 5.1 for Cal 27). The tumor-to-background ratio (contrast) allowed precise fluorescent delineation of the tumor in all xenografted animals. Control tongues had an average radiance efficiency signal significantly lower (*P* < 0.001) than xenografted tongues (Supplementary Fig. 3).

Further exploring the potential of PARPi-FL to guide surgical decision-making, we modeled partial glossectomies in mice. These partial glossectomies contained margins that were intentionally compromised. Animals tongues were imaged with both the IVIS epifluorescence and the Lumar fluorescence stereoscope. Widening of surgical margins was performed until no fluorescent signal could be observed and results were compared against standard H&E histology. PARPi-FL was able to identify all compromised margins by means of its fluorescent emission. Margins free of disease presented with almost no signal in both IVIS (Fig. 2b) and stereoscope (Fig. 2c). We found that PARPi-FL was able to differentiate tumor, compromised, and margin-negative resections in all animals (Fig. 2d).

### Hand-held confocal microscopy can identify xenografted tumor cells

**Figure 3:**
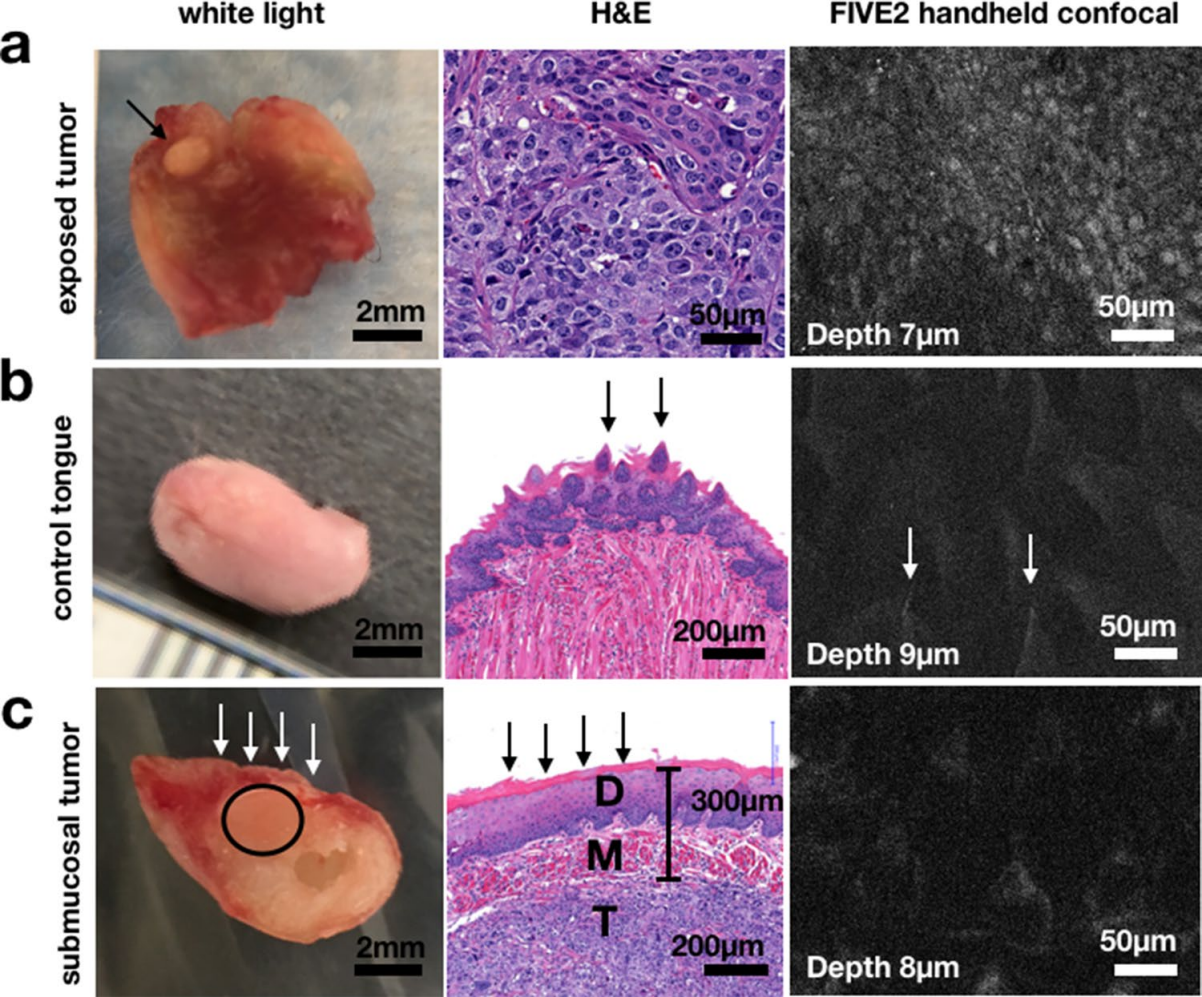
Imaging of orthotopic tongue tumors using a hand-held point scanner confocal microscope for microscopic identification of tumors cells. FIVE2 image settings: BP 515–575, brightness 94–99%, frame rate: 1/s. (**a**) Interrogating an exposed lesion, the imaging system was able to identify the small island of tumor within the normal tongue muscle. The signal was specific and arising from the nuclei of tumor cells, where PARP1 is expressed. (**b**) In healthy tongues of mice also injected with PARPi-FL, no nuclear signal was identified, while autofluorescence from the papillae was seen, but can be easily differentiated from the specific signal observed in tumors. (**c**) No nuclear signal was picked up through the outer mucosal and basal layers of the mouse tongues. Tumor cells were observed on H&E-stained histological slides within the musculature (in this case at 300 µm depth). The representative image shows an example taken at 8 µm depth; no specific PARPi-FL signal could be detected aside from tongue’s papillae. T = Tumor, M = Healthy Muscle, D = Healthy Dermis and basal layer.

To illustrate that PARPi-FL is also suitable for contemporaneously assessing margins at a cellular level, we tested its application with a hand-held confocal microscope (Supplementary Fig. 4). In an exposed lesion of an orthotopic mouse xenograft (FaDu), PARPi-FL gave rise to a bright specific signal that could be detected by confocal microscopy, whereas no specific signal could be seen in the normal tongue muscle cells of the same specimen (Fig. 3a). Similarly, in healthy tongues of mice also injected with PARPi-FL, no specific signal could be identified. Mice tongues are very keratinized; therefore, autofluorescence from the papillae could be responsible for the non-nuclear, more diffuse signals observed, but can be easily differentiated from the specific signal seen in tumor (Fig. 3b). In mice, identification of submucosal PARPi-FL signal through the outer heavily keratinized epithelium was not possible without surgical exposure. This is particularly relevant for the orthotopic tumor xenografts, which are injected into the mouse muscle and consequently originate in the deep tissue layers (Fig. 3c). Importantly, this was not a limitation when the tumor surface was exposed, which is typically the case during surgical resection (Fig. 3a).

### Chemically induced tumors are similar to human squamous cell carcinoma

**Figure 4:**
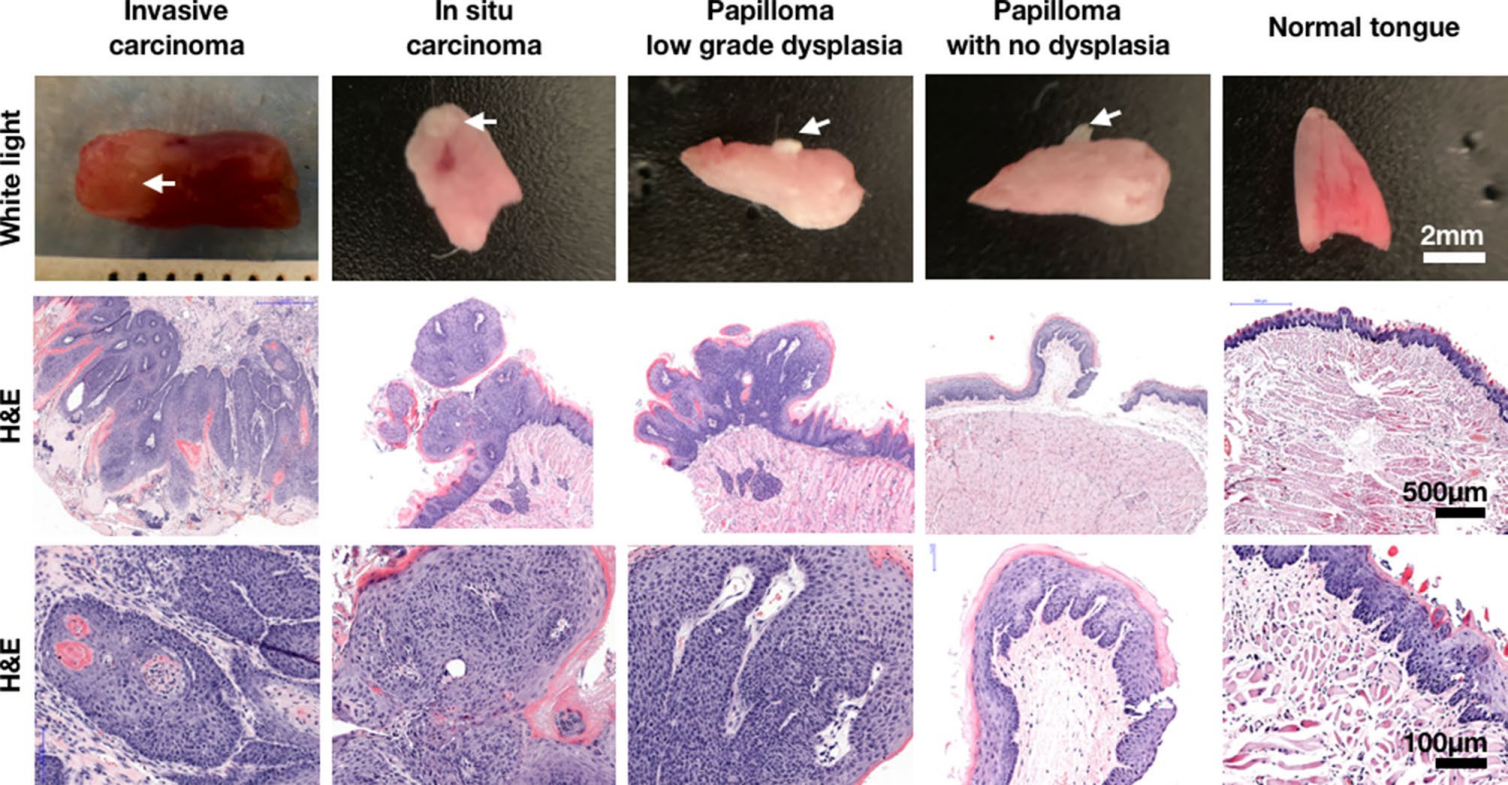
Physiological representation of chemically induced oral lesions in C57BL/6J black mice. First row: phenotypical presentation of mouse tongues after resection; second row: H&E slide at lower magnification; third row: zoomed-in image of the H&E slide (higher magnification).

Chemically induced oral tumors presented a much more complex physiological environment than xenografts, ranging from no tumor to papilloma with no dysplasia, papilloma with low grade dysplasia, in situ carcinoma and invasive squamous cell carcinoma (Fig. 4). Intuitively, this mouse model better mimics the human phenotype, as it recapitulates carcinogenesis and features field cancerization of the exposed mucosa. To induce tumors, a solution of 4-Nitroquinoline 1-oxide (4NQO) in propane-1,2-diol was topically administered three times a week. Much like in humans, some animals developed tumors earlier than others—as early as 16 weeks in one case, while others did not develop tumors even after 50 weeks (the experimental endpoint). On average each mouse received 127 ± 30 applications. Out of 25 mice, 19 (76%) developed oral or oropharyngeal lesions, whereas only six (24%) had no lesions. For the six mice that did not develop oral lesions, one mouse died of an unknown cause in Week 16 (no oral/oropharyngeal or gastrointestinal tumor was found during necropsy) and one mouse was euthanized in Week 29 due to the development of a bleeding lesion on its left paw. Out of the 19 mice with lesions, 37% (n = 7) developed papilloma/oral dysplasia (4 papilloma, 3 low-grade dysplasia and none with moderate- or high-grade), 11% (n = 2) developed carcinoma in situ and 42% (n = 8) developed invasive squamous cell carcinoma. Because of the experimental design, most of the lesions (n = 16) developed in the oral tongue. We had one oral tumor that developed in the floor of mouth and one oropharyngeal tumor (base of tongue). Two mice with lesions did not have their tongues removed for histopathological analysis.

### Hand-held confocal microscopy can identify tumor cells in chemically induced tumors

**Figure 5:**
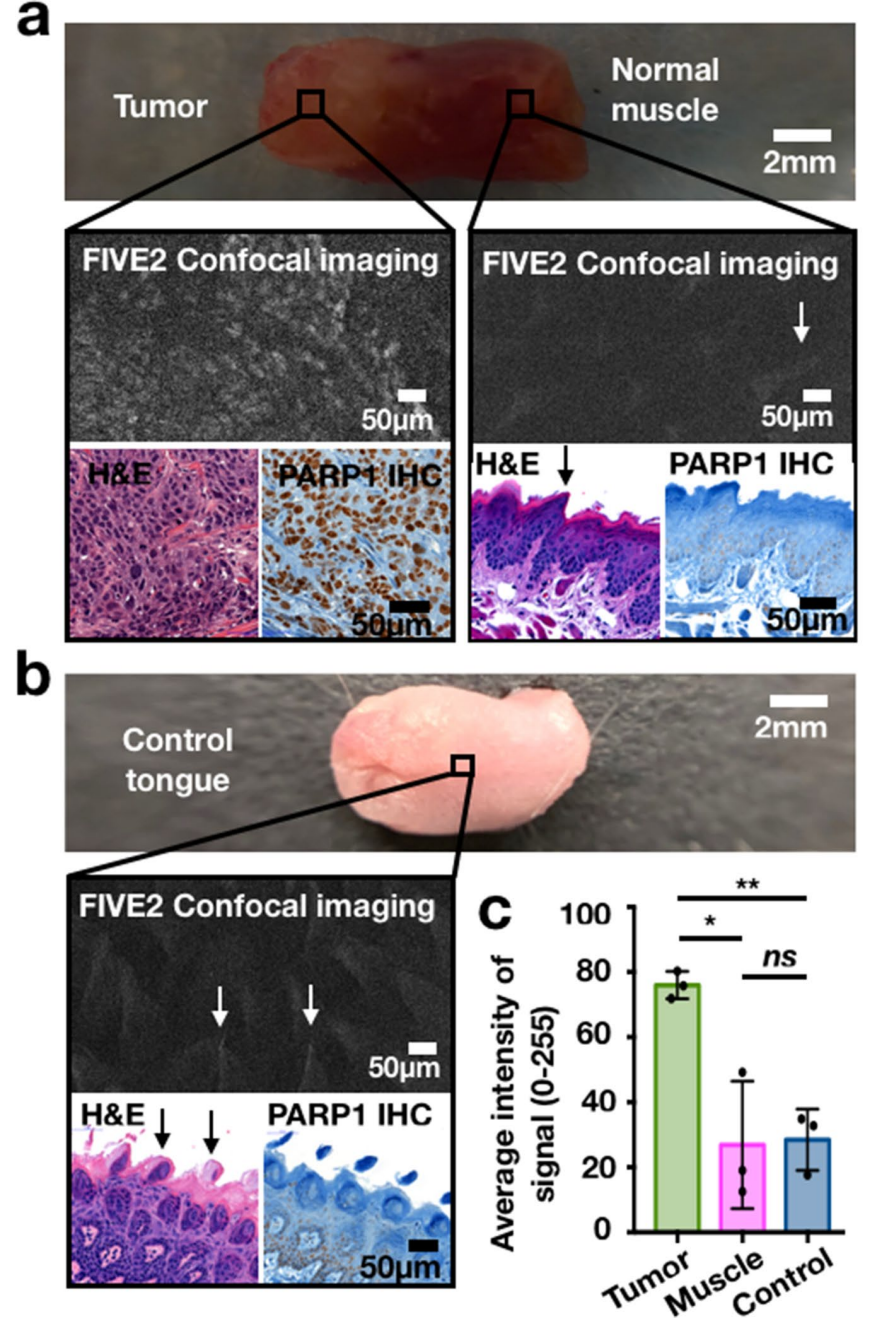
Hand-held confocal imaging in mice with chemically induced tumors. Mice were intravenously injected with 0.043 mg/kg of PARPi-FL and sacrificed after 90 min; excised tongues were then immediately imaged. Because this is a chemically induced mouse model, tumors grow in the tongues’ mucosal surface. FIVE2 image settings for tumor, margins and control: BP 515–575, brightness: 94%, frame rate: 1/s, 24 µm depth. (**a**) PARPi-FL signal indicated presence of tumor tissue, corroborated with PARP1 IHC and H&E. No nuclear signal was observed for healthy surrounding tissue, and absence of tumor was confirmed with PARP1 IHC (faint limited staining compared to tumor) and H&E. (**b**) No nuclear signal was observed in the control tongue, and absence of tumor was confirmed with H&E and PARP1 IHC (faint, limited staining compared to tumor). Autofluorescence from the normal keratin found in mice gustative papillae was observed. (**c**) The average signal intensity arising from the control tongue was not significantly different (*P* > 0.99) from the normal muscle around the tumor, whereas the average signal intensity generated by the tumor areas (76.0 ± 4.2 a.u.) was significantly higher when compared to the normal adjacent tongue muscle (27.0 ± 19.6 a.u., *P* < 0.05) and to control (average intensity: 28.4 ± 9.4 a.u., *P* < 0.01).

The chemically induced oral cancers exhibited fundamentally different and more phenotypically accurate growth patterns than the orthotopic tumors, which resulted in tumor cells being located much closer to the tissue surface. Non-invasive identification of tumors was therefore possible using the FIVE2 confocal fluorescence microscope where tumor cells were distinguishable from normal surrounding tissue and healthy mouse tongue (Fig. 5). Analogous to our findings with orthotopically injected xenografts, we saw only unspecific signals corresponding to the autofluorescence arising from the tongue’s papillae in normal muscle and control tongues. The signal distribution pattern for autofluorescence is not nuclear and is distinct from specific signals arising from the nuclei of tumor cells. In addition, while the average signal intensity arising from the control tongue did not significantly differ (*P* > 0.05) from the normal muscle surrounding the tumor, the average signal intensity generated by the tumor areas (76.0 ± 4.2 a.u.) was significantly higher than normal adjacent tongue muscle (26.9 ± 19.6 a.u., *P* < 0.05) and control (*P* < 0.05, average intensity: 28.4 ± 9.4 a.u., *P* < 0.01; Fig. 5c). Presence of tumors was further corroborated by H&E histology and PARP1 IHC, which confirmed that the PARPi-FL signal correlated to tumoral expression of PARP1.

**Figure 6:**
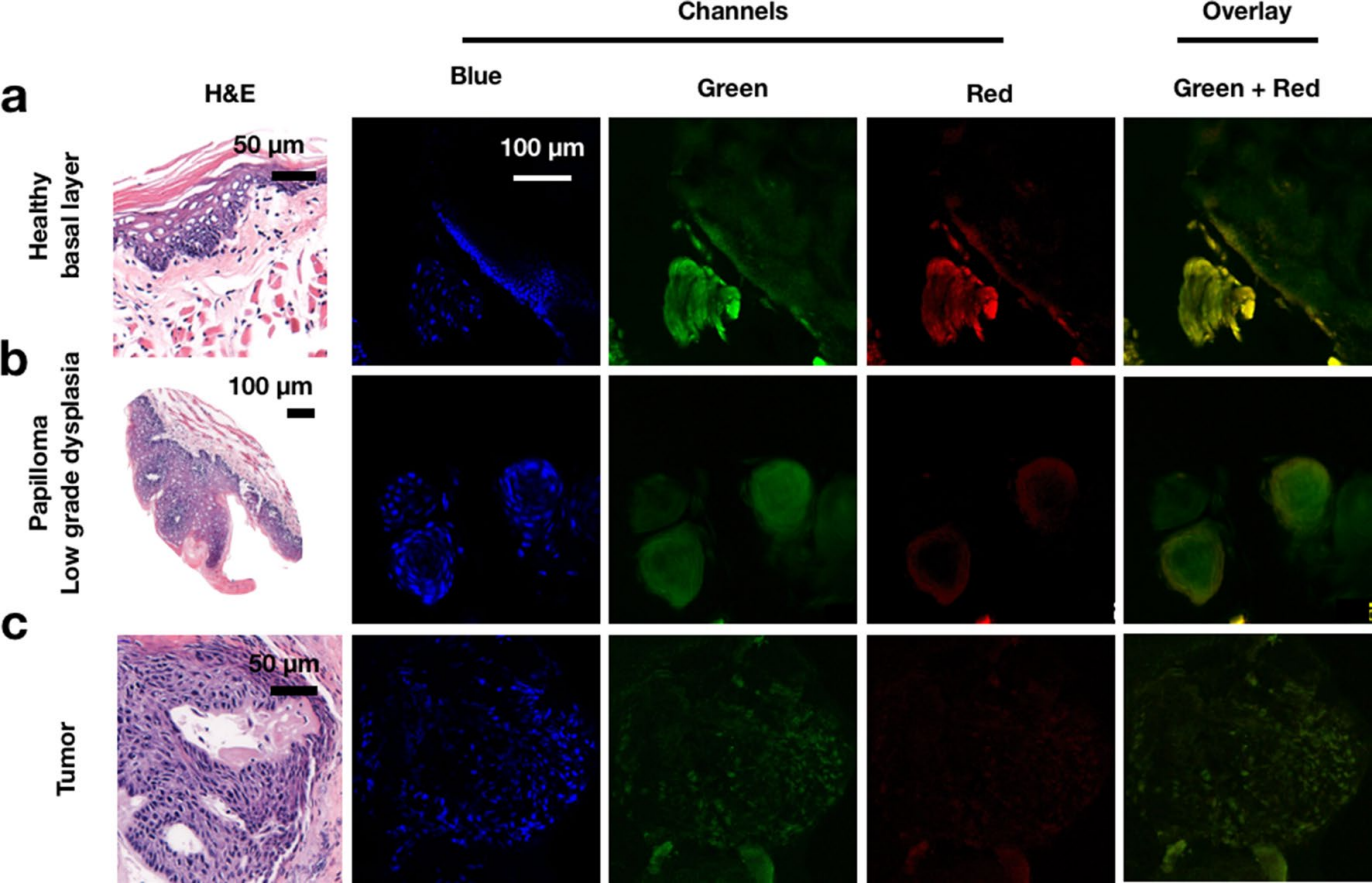
Histological analysis of PARPi-FL uptake in C57BL/6J mice with chemically induced oral lesions. (**a**) Confocal microscopy showed minimal specific nuclear PARPi-FL signal arising from tongues without lesions. Nuclei of the basal layer cells showed Hoechst 33,342 uptake, but not PARPi-FL. Most of the detected signal in the green channel was demonstrated to be autofluorescence (red channel). (**b**) Confocal microscopy also demonstrated little specific nuclear PARPi-FL signal arising from tongues with low-grade dysplasia. (**c**) PARPi-FL was able to identify tumor cells, showing strong overlapping signal with the Hoechst 33,342 channel, but not the red autofluorescence channel.

Additionally, regular confocal microscopy was also carried out in the various chemically induced lesions. Nuclei of basal layer cells were clearly visible with Hoechst 33,342 DNA stain but showed only a very faint signal with PARPi-FL (Fig. 6a). The same findings were observed when a papilloma harboring low-grade dysplasia was imaged. Most of the signal originating in the green channel was also present in the red channel and was therefore attributed to autofluorescence, likely due to keratin, which is commonly found in papilloma (Fig. 6b). However, when imaging an invasive squamous cell carcinoma, we found a strong specific PARPi-FL nuclear-associated signal in the green channel. The signal matched the DNA stain, suggesting that it was a specific nuclear uptake. A minimal amount of autofluorescence was detected in those images (Fig. 6c).

## Discussion

In this study, we have demonstrated the capability to achieve contemporaneous and specific visualization of compromised margins in OSCC models in mice using the fluorescent contrast agent PARPi-FL. This approach has the potential to improve the identification of tumor-positive resection margins during surgery, which are associated with lower survival rates^9^. By reducing required margin widths, it can also help clinicians reduce the amount of healthy tissue resected, leading to significant improvements in patients' quality of life^4^.

Several contrast agents have been or are still being explored as tools to help surgeons overcome this problem: these include fluorescent imaging agents (such as γ‐glutamyl hydroxymethyl rhodamine green^27^, indocyanine green^28^, urokinase‐like plasminogen activator receptor^29^ cetuximab/panitumumab-IRDye800^30^) and non-fluorescent dyes (for example Lugol's iodine^31^ and toluidine blue^32^). However, the gold standard today remains ex vivo histopathological assessment of paraffin-embedded sections. A major limitation of this approach to margin assessment is that paraffin-embedded sections are not only non-contemporaneous, they are also subject to specimen shrinkage and post-operative anatomical deformations of the tumor bed^33^. The specimen shrinkage phenomenon, especially after processing the tissue for paraffin embedding, is well recognized. A classic study, conducted on a canine model, showed a mean shrinkage of up to 47.3% on labiobuccal mucosal margins^34^. This situation makes it very difficult to accurately correlate the location of a positive margin on the ex vivo specimen to its in situ counterpart, where it needs to be removed. Intuitively, analyzing the tumor bed itself for compromised margins would ameliorate this issue.

PARPi-FL is efficient in targeting PARP1 within the nucleus of cells, since the modification is made on the cyclopropane end of the olaparib scaffold—a region of the PARP inhibitor that is not essential for target binding^19,21,22^. Other dyes, including those in the NIR region, were explored by us as well, but didn’t provide sufficient in vivo performance^35^.

Using whole-mouse tissue sections, we corroborated that PARPi-FL accumulates in the tumor and in neck metastases, with low signal in normal head and neck tissues (Supplementary Figs. 1, 2). Although autofluorescence was also detected in the green emission fluorescence channel, it is important to emphasize that this can be removed during post-processing due to differences in peak width when comparing autofluorescence and PARPi-FL^18,36^. Imaging agrees with previous literature findings, where PARPi-FL excretion was observed to be hepatobiliary (Supplementary Fig. 2)^37,38^.

When intravenously injected, PARPi-FL penetrated the full depth of the tumor, the neck metastasis and the small lesions induced by 4NQO. When considering margin assessment, tumor delineation, and in vivo detection of compromised margins, intravenous injection offers great advantages over topical application^15,18,36^. Its tissue penetration enables true real-time surgical guidance and post-resection intraoperative assessment of all surgical margins by scanning the tumor bed both macro- and microscopically. Macroscopic imaging enables precise tumor delineation, which could be used for guided surgical resection. PARPi-FL would also be capable of ensuring negative-margin resection based on an absence of signal in the tumor bed after resection (Fig. 2). Histology corroborated that no tumors were found in tissues with absence of signal, whereas those with fluorescence harbored tumor cell deposits (Fig. 2b, c). Using a hand-held confocal microscope, we were also able to contemporaneously and non-invasively visualize margins at cellular resolution, differentiating tumors from healthy cells (Figs. 3, 5).

The impact of moderate and high-grade dysplastic margins around the tumor bed area on overall survival is somewhat unclear^39,40^, although it’s known that no association exists between low-grade dysplasia and higher recurrences or lower survival rates^40^. Therefore, excising an area of low-grade dysplasia adjacent to an invasive cancer can negatively affect quality of life, since large resections generally result in worse functional outcome. Interestingly, we found that PARPi-FL was picked up only to a limited degree by the low-grade dysplastic tissues in mouse models, suggesting that this small molecule can be used to reliably assess tumor margins even when the oral cavity harbors other types of dysplasia (Fig. 6). All signal seen in the green channel of normal and dysplastic tissues was morphologically different from that seen in tumor. This signal was not specific to the cells' nuclei, and matched with the signal obtained in the red channel (autofluorescence). Only in tumor was a strong correlation between PARPi-FL and Hoechst observed, suggesting the presence of a specific nuclear signal.

In this study, we used three different types of mouse models: two orthotopic xenograft models (using FaDu and Cal 27 cells) and one with chemically induced tumors. An important consideration is that while xenografts develop much faster than chemically induced tumors, tumor cells grow from the center of the tongue towards the surface, leaving the basal layer initially undisturbed. On the other hand, chemically induced tumors recapitulate the human phenotype much more closely. This model is based on the repeated topical application of 4NQO. Metabolites of 4NQO bind to nucleic acids, predominantly at guanine residues, creating damage that resembles that imposed by other carcinogens present in tobacco^41,42^. 4NQO-induced tumors for this mouse model grew much more diffusely and originated in the mucosal layers of the tongue, much like those observed in humans. In addition, 4NQO lesions exhibit similar histological and molecular changes as the cancers observed in human oral carcinogenesis^43,44^. C57BL/6J black mice were used as a background for this mouse model because, unlike most mouse strains, C57BL/6J black mice drink alcoholic beverages voluntarily^45^, facilitating the delivery of the 4NQO, which was dissolved in propane-1,2-diol. Importantly, irrespective of the mouse model, PARP1 was reliably overexpressed in all tumors and intraoperative delineation of tumor versus normal tissue was consistently possible using PARPi-FL.

For future directions, especially when considering clinical use, it is important to emphasize that PARPi-FL is a green fluorescent small molecule with an emission spectrum similar to FITC. Due to its green fluorescence, both PARPi-FL and autofluorescence emission spectra overlap. Nonetheless, the specific signal (PARPi-FL) can be separated from the non-specific (autofluorescence) emissions due to the broader autofluorescence peak width. This is being done for an ongoing clinical trial using topical application of PARPi-FL for detection of oral cancer^18^.

Finally, consideration of dosing is important towards clinical translation. A typical therapeutic dosing regimen of olaparib is 600 mg/day given orally for up to 2 years^46,47^. This corresponds to 8.6 mg/kg assuming an average weight of 70 kg. The dose of PARPi-FL used in this study is 48 µg or 1.9 mg/kg per mouse (for a typical 20 g mouse). Using an extrapolation formula to account for the metabolic differences and body surface area between human and mouse^48^, the concentration translates to a human equivalent dose (HED) of 0.15 mg/kg. This corresponds to a single 10 mg injection for a 70 kg adult, which is 60-times lower than the daily dose of olaparib. Importantly, in a recently published study, PARPi-FL could be detected in a large animal imaging experiment at the dose level of 0.05 mg/kg^18^, which is lower than the 0.15 mg/kg calculated here and supports the hypothesis that the HED of PARPi-FL can generate a suitable in vivo contrast for cancer detection in humans.

## Conclusion

PARPi-FL was able to delineate oral squamous cell carcinoma both macro- and microscopically, differentiating tumor from dysplasia and identifying compromised margins. Intravenously injected PARPi-FL has great potential for clinical translation because of its ability to help surgeons assess oral cancer margins in vivo during surgery.

## Materials and methods

### General methods

The study demonstrates the use of PARPi-FL to delineate oral tumors in 2 different oral cancer mouse models, a xenografted, using FaDu and Cal 27 cell lines, and a chemically induced model, using the chemical 4-nitroquinolone-1-oxide—4NQO. For the experiments, animals with tumor-bearing tongues and controls were injected intravenously via tail vein with 48 µg of PARPi-FL per animal, dissolved in 30% PEG300 in PBS, and euthanized after 90 min. In total 59 mice were utilized. In the xenograft group of experiments a total of 15 animals were used (11 xenografted with FaDu and 4 with Cal 27 cell line). For the induced group, 25 mice had their tongues exposed to 4NQO. Nineteen mice were used as controls. Different imaging techniques were used and are described below. All reactions were magnetically stirred, and room temperature refers to 20–25 °C. High-performance liquid chromatography (HPLC) purification and analysis was performed on a Shimadzu UFLC HPLC system equipped with a DGU-20A degasser, an SPD-M20A UV detector, a LC-20AB pump system, and a CBM-20A communication BUS module. HPLC solvents—Buffer A: 0.1% Trifluoroacetic Acid (TFA) in water, Buffer B: 0.1% TFA in Acetonitrile (MeCN)—were filtered before use. HPLC purification and analysis of PARPi-FL was performed on an analytical column reversed-phase Atlantis T3 5 µm column (C18, 4.6 mm, and 250 mm). Purification and analysis of PARPi-FL: flowrate—1.0 mL min^−1^; gradient—0–15 min 5–95% of buffer B; 15–18 min 95% of buffer B; 18–18.3 min 95–5% of buffer B. An automated cell counter (Beckman Coulter, Vi-Cell viability analyzer) was used for counting the number of cells. Fluorescence confocal microscopy on tissues were carried out using a LSM880 Airyscan confocal microscope (Zeiss, Germany) or FIVE2 handheld confocal microscope (Optiscan, Australia).

### PARPi-FL synthesis

#### Chemicals

Commercially available compounds were used without further purification unless otherwise stated. Bio Ultra Polyethylene Glycol (PEG) 300, Ethyl 4-nitrobenzoate, potassium carbonate (K_2_CO_3_), triethylamine (NEt_3_) trifluoroacetic acid (TFA) and 4-nitroquinolone-1-oxide (4NQO) were purchased from Sigma-Aldrich (St. Louis, MO). HPLC and Liquid Chromatography-Mass Spectrometry (LC–MS) grade MeCN were obtained from Fischer Scientific (Hampton, NH). Water (> 18.2 MΩ cm^−1^ at 25 °C) was obtained from an Alpha-Q Ultrapure water system from Millipore (Bedford, MA). 4-(4-Fluoro-3-(piperazine-1-carbonyl)benzyl)phthalazin-1(2*H*)-one (PARP-NH precursor) was purchased from AA blocks (San Diego, CA) and purified by HPLC using the method described previously in general methods before its use and further synthesis. BODIPY-FL NHS-ester was purchased from Invitrogen, Carlsbad, CA without further purification. PARPi-FL was kept as a 1.5 mM stock solution in BioUltra PEG 300 and diluted to the final working concentration for the respective experiments.

#### Synthesis

PARPi-FL was synthesized according to our previously described procedure^20,23,37^. Briefly, fluorescent dye BODIPY-FL NHS-ester (1.0 equivalent) was conjugated to 4-(4-fluoro-3-(piperazine-1-carbonyl)benzyl)phthalazin-1(2*H*)-one (1.0 equivalent) under a base, Et_3_N (5.0 equivalents) in MeCN for 4 h at room temperature. It was purified by preparative HPLC (Atlantis T3 5 μm column 4.6 × 250 mm, 1 mL/min, 5 to 95% of acetonitrile in 15 min) to afford PARPi-FL in 70–79% yield as a red solid. Analytical HPLC analysis (Waters’ Atlantis T3 C18 5 μm 4.6 × 250 mm column) showed high purity (> 99%, t_R_ = 13.9 min) of the imaging agent. The identity of PARPi-FL was confirmed using Electrospray Ionization Mass Spectrometry (MS( +) *m/z* = 663.63 [M + Na]^+^).

### Mouse models

#### Animals

**Figure 7:**
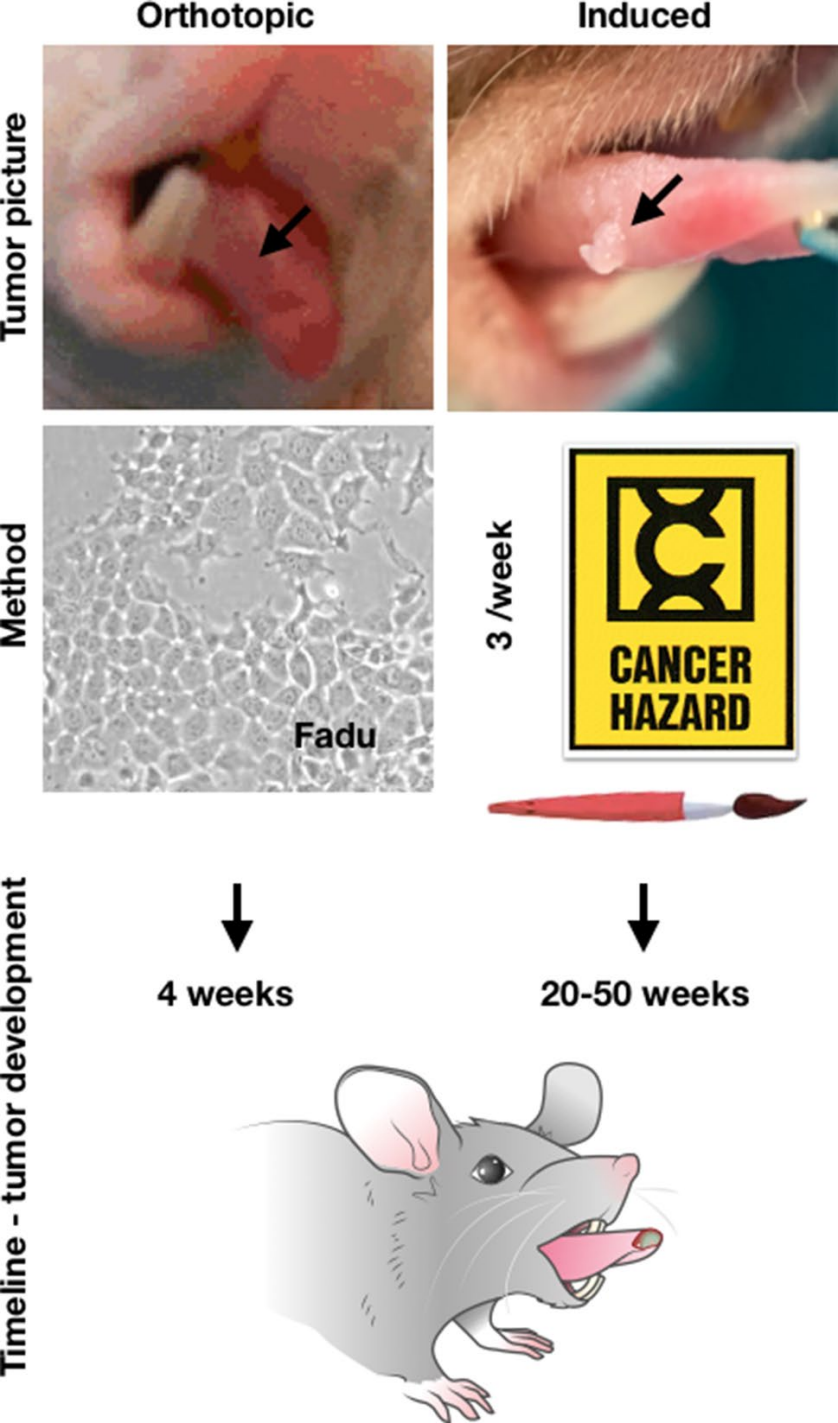
Orthotopic and chemically induced mouse models of squamous cell carcinoma used in the project. To create an orthotopic mouse model, athymic nude mice were xenografted with 500,000 cells of an oral squamous cell carcinoma cell lines (FaDu or Cal 27). An advantage of this model is that tumors grow faster (4 weeks). On the other hand, cells are injected into the tongue, causing tumors to grow from the muscle to the surface and leaving the basal layer and the dermis of the tongue intact. Additionally, no pre-cancerous lesions develop. To generate chemically induced oral lesions, 4NQO in propane-1,2-diol was applied to mice tongues 3 times a week (average of 127 ± 30 applications per mouse). The advantage of this model is that tumors develop much like in humans, within a much more complex physiological environment, leading to the development of a variety of lesions ranging from benign papilloma to invasive carcinoma in the mouse oral mucosa. In this experiment, nineteen mice (76%) developed oral or oropharyngeal lesions. Nevertheless, oral lesions using this model take a much longer time to develop, and other tumors (e.g. gastrointestinal tract tumors) are also likely to appear. Besides this, the true nature of the developed lesion (e.g. invasive OSSC, in situ carcinoma, dysplasia) can only be confirmed after final histological confirmation.

All animal experiments were performed in accordance with protocols approved by the Institutional Animal Care and Use Committee (IACUC) of MSK and followed the National Institute of Health guidelines for animal welfare. For the xenograft group, athymic nude mice (Envigo, Somerset, NJ) were inoculated on the 1/3 anterior and ventral portion of the right side of the tongue with 500,000 cancer cells diluted in 20 µL of PBS and allowed to grow for 4 weeks. One mouse was xenografted with fewer cancer cells (250,000 FaDu cells) and a 10-week growth period, in order to have a slower tumor growth and allow detectable neck metastases to develop. In this case, tumor and neck metastases were allowed to grow for 10 weeks. For the chemically induced group (n = 25), C57BL/6J black mice (Jackson laboratory, Farmington, CT) were lightly anesthetized by inhalation of isoflurane (at 2% of isoflurane in oxygen 2L/min using a vaporizer) and the previously described solution of 4NQO was applied with a single stoke to the tongue with a Number 2 paint brush, which was reported to deliver a relatively constant carcinogen quantity of 0.15 ± 0.03 mg per application^42,44^ for a maximum of 50 weeks (3 times weekly, maximum of 150 applications per mouse), whereupon all mice were euthanized irrespective of tumor development (Fig. 7). All animals were examined weekly to observe body weight loss, tumor growth (tumor development was assessed by visual inspection of the entire oral cavity with the mice under isoflurane anesthesia) and possible development of metastases. Mice were also euthanized in case of body weight loss (> 20%), presence of large or ulcerated tumors that would impair the animals' ability to eat or additional signs of suffering.

#### Cell lines and cell culture for the xenografted mouse model

FaDu and Cal 27 cell lines were purchased from ATCC, Manassas, VA. Cells were grown in a monolayer culture at 37 °C in a 5% CO_2_ humidified atmosphere. FaDu cells were maintained in Minimal Essential Media (MEM) and Cal 27 in Dulbecco’s Modified Eagle Media (DMEM). Both mediums contained 10% (v/v) fetal bovine serum (FBS) and 1% (v/v) PenStrep. Both cell lines tested negative for mycoplasma infection. FaDu is a squamous cell carcinoma cell line derived from an Indian men harboring a squamous cell carcinoma of the hypopharynx^49^. Cal 27 is derived from a 56-year-old caucasian male harboring a poorly differentiated squamous cell carcinoma of the oral tongue^50,51^.

4-Nitroquinolone-1-oxide (4NQO) solution preparation for the chemically induced mouse model. Batches of the carcinogen 4-nitroquinolone-1-oxide (Sigma-Aldrich,St. Louis, MO) were divided into 50 mg aliquots sealed and stored in a − 20 °C refrigerator until used. At regular intervals fresh solutions of carcinogen were prepared at 0.5% w/v concentration in propane-1,2-diol and stored in a + 4 °C refrigerator for further application^43,52^.

#### Obtaining surgical compromised margins

Compromised margins were obtained by performing glossectomies and intentionally leaving small tumor tissue in the main tongue specimen. Widening of surgical margins were performed until no macroscopic tumor could be observed. Results were further compared against histology to ensure that the macroscopic results were accurate.

### Imaging

#### Whole-mouse white light and fluorescence imaging

A mouse with oral cancer and palpable bilateral neck metastases along with a control mouse were imaged to evaluate PARPi-FL uptake. Ninety minutes post-intravenous tail vein injection of PARPi-FL, mice were euthanized and flash-frozen by placing them into a hexanes/dry-ice-filled dewar for 10 min. Both mice were sent to Emit (Boston, Ma, USA) for imaging. Briefly, both samples were further embedded in optimal cutting temperature compound and sectioned (one slice per 50 µm). All slides were imaged using a high-throughput whole-body sectioning and imaging system (Xerra, EMIT imaging, Boston, MA, USA) with a 470 nm excitation laser (emission filter: 511/20, average exposure: 288 ms) and white light. Images from each scanned slice were overlaid for assessment and further reconstructed into a 3D structure, so the data could be visualized in multiple orientations.

#### Epifluorescence imaging

PARPi-FL was detected using the IVIS SPECTRUM/CT (PerkinElmer, USA) fluorescence detector with a pre-defined GFP filterset (excitation: 465/30 nm, emission: 520–580 nm). Autofluorescence was subsequently removed through spectral unmixing. Semiquantitative analysis of the PARPi-FL signal was conducted by measuring the average radiant efficiency in regions of interest (ROIs) placed in tumors and normal tongues. These measures carried the unit [p/s/cm^2^/sr]/[µW/cm^2^]. Variables, including the integration time, binning, f/stop, field of view, and illumination intensity were kept constant across experiments.

#### Stereoscope imaging

Images were acquired on a Zeiss Stereo Lumar stereoscope, equipped with a Hamamatsu Orca-ER CCD camera and a GFP filterset (excitation: 470/40 nm, emission: 525–50 nm), controlled with the micromanager v1.14 software. Fluorescence images were collected using a 1.5 × magnification objective and exposure time of 236 ms in all groups. Images were further processed with Fiji (Image J), keeping the same settings for tissues within the same group. Because of differences in tissue thickness and tumor sizes, the settings were different for each group (different for each mouse).

#### Handheld confocal fiberoptic imaging

Images were acquired using the FIVE2 (Optiscan, Victoria, Australia) by using a single channel for illumination and detection of PARPi-FL (488 nm excitation) and lens NA = 0.3 (similar to a 10 × objective) with a field of view of 475 µm × 475 µm. The probe was in direct contact with the tissue to acquire images. Different depths were assessed by using the instrument’s Z mechanism (Z axis, 0 µm–400 µm). For all experiments, we used a band pass filter of 515–575 nm, 94–100% laser power %, and a scanning speed of 1 frame per second. The depth at which the image was acquired is specified in each image.

#### Tabletop confocal microscopy

Fresh tongue (whole-mount) tissues of mice intravenously injected with PARPi-FL were further stained with a solution 10 µg/mL solution of Hoechst 33,342 in PBS and placed over a cover slip glass slide (48 × 60 mm no. 1 thickness, Brain Research Laboratories, Newton, MA). Images were acquired with a laser scanning confocal microscope (LSM880-Live, Zeiss, Germany) using 488 nm laser excitation for PARPi-FL (green), 405 nm for Hoechst (blue) and 561 nm (red) for autofluorescence.

### Tissue processing and histology

#### PARP1 immunohistochemistry

PARP1 IHC was performed following our previously published protocol^15^. Paraffin-embedded slides were processed at the molecular cytology core facility at MSK for processing. PARP1 immunohistochemistry was performed by an automated system using the Discovery XT processor (Ventana Medical Systems, Tucson, AZ). The anti-PARP1 rabbit monoclonal antibody (46D11, Cell Signaling Technology, Danvers, MA) specifically bound both human and mouse PARP1 (0.4 μg/mL). Paraffin-embedded formalin fixed 3 μm sections were deparaffinized with EZPrep buffer, antigen retrieval was performed with CC1 buffer (both Ventana Medical Systems, Tucson, AZ), and sections were blocked for 30 min with Background Buster solution (Innovex, Richmond, CA). Anti-PARP1 antibody was incubated for 5 h, followed by 1 h of incubation with biotinylated goat anti-rabbit IgG (PK6106, Vector Labs, Burlingame, CA) at a 1:200 dilution. For IHC detection, a DAB detection kit (Ventana Medical Systems, Tucson, AZ) was used according to the manufacturer’s instructions, sections were counterstained with hematoxylin and cover-slipped with Permount (Fisher Scientific, Pittsburgh, PA). Incubating with a rabbit IgG instead of the primary antibody controlled for non-specific binding of the secondary antibody. Slides were scanned (Mirax, 3DHISTECH, Budapest, Hungary) to allow for digital histological correlation. H&E stained slides were used to determine areas of tumor and areas of normal muscle. Those exact same areas were used for PARP1 quantification using an immediately consecutive slide. Quantification of PARP1 was carried out on digitalized slides. Thresholding was performed (Fiji, Image J) on brown (PARP1 marked with 3,3′-Diaminobenzine—DAB) and blue (tissue) areas and the relative PARP1-positive area was calculated by dividing the brown (DAB) area by the blue (total tissue area). The entire tongue tissue was encompassed for analysis.

#### H&E staining

Specimens were fixed in 4% paraformaldehyde (PFA, MP Chemicals, Solon, OH) in sterile water for 12 h at 4 °C and kept for 12 h in 70% ethanol and sent to the MSK Molecular Cytology Core facility. Paraffin embedded sections were obtained and further stained with hematoxylin and eosin (H&E) by the MSK Molecular Cytology Core facility. Slides were scanned (Mirax, 3DHISTECH, Budapest, Hungary) to allow for digital histological correlation.

### Data analysis

#### Statistical analysis

Statistical analysis was performed using GraphPad Prism 7. Data points represent mean values, and error bars represent standard deviations. We used the Wilcoxon–Mann–Whitney test to compare the differences between the groups. Statistical significance was determined with alpha = 0.05 and the level of significance for each result displayed as *P < 0.05, **P < 0.01, ***P < 0.001 and ****P < 0.0001.

## Supplementary information

Supplementary file1

## Abbreviations

PARP1: Poly (ADP ribose) polymerase1
PARPi: PARP1-poly (ADP ribose) polymerase1 inhibitor
millimeters: mm
OSSC: Oral squamous cell carcinoma
HPV: Human papilloma virus
a.u.: Arbitrary units
MSK: Memorial Sloan Kettering Cancer Center
H&E: Hematoxylin and eosin stain
IHC: Immunohistochemistry
HPLC: High-performance liquid chromatography
TFA: Trifluoroacetic Acid
MeCN: Acetonitrile
PEG: Polyethylene glycol
LC–MS: Liquid chromatography–mass spectrometry
PARP-NH precursor: 4-(4-Fluoro-3-(piperazine-1-carbonyl)benzyl)phthalazin-1(2*H*)-one
MEM: Minimal Essential Media
DMEM: Dulbecco’s Modified Eagle Media
FBS: Fetal bovine serum
DAB: 3,3′-Diaminobenzine
PFA: Formaldehyde
