# Supplementary material for: Fluorescence-guided resection of tumors in mouse models of oral cancer
Source: Sci Rep. 2020 Jul 7;10:11175. doi: 10.1038/s41598-020-67958-8 (PMC7341853; doi:10.1038/s41598-020-67958-8)
Supplement: Supplementary file 1 — Supplementary file1 [file 41598_2020_67958_MOESM1_ESM.docx]

SUPPLEMENTARY MATERIAL TO:

**Fluorescence-guided resection of tumors in mouse models of oral cancer.**

Paula Demétrio De Souza França^a,b^, Navjot Guru^a^, Sheryl Roberts^a^, Susanne Kossatz^a,c^, Christian Mason^a^, Marcio Abrahão^b^, Ronald A. Ghossein^d^, , Snehal G. Patel^e,f^, Thomas Reiner^a,f,g*^

^a^ Department of Radiology, Memorial Sloan Kettering Cancer Center, New York, NY, USA.

^b^ Department of Otorhinolaryngology and Head and Neck Surgery, Federal University of São Paulo, SP, Brazil.

^c^ Department of Nuclear Medicine, School of Medicine, Technische Universität München, Munich, Germany.

^d^ Department of Pathology, Memorial Sloan Kettering Cancer Center, New York, NY, USA.

^e^ Department of Surgery, Memorial Sloan Kettering Cancer Center, New York, NY, USA.

^f^ Weill Cornell Medical College, New York, NY, USA.

^g^ Chemical Biology Program, Memorial Sloan Kettering Cancer Center, New York, NY, USA.

Correspondence should be addressed to:

*Thomas Reiner

1275 York Avenue

New York, NY, 10065

[reinert@mskcc.org](mailto:reinert@mskcc.org); (P) 1-646-888-3461; (F) 646 442 0408

**Figures**

**
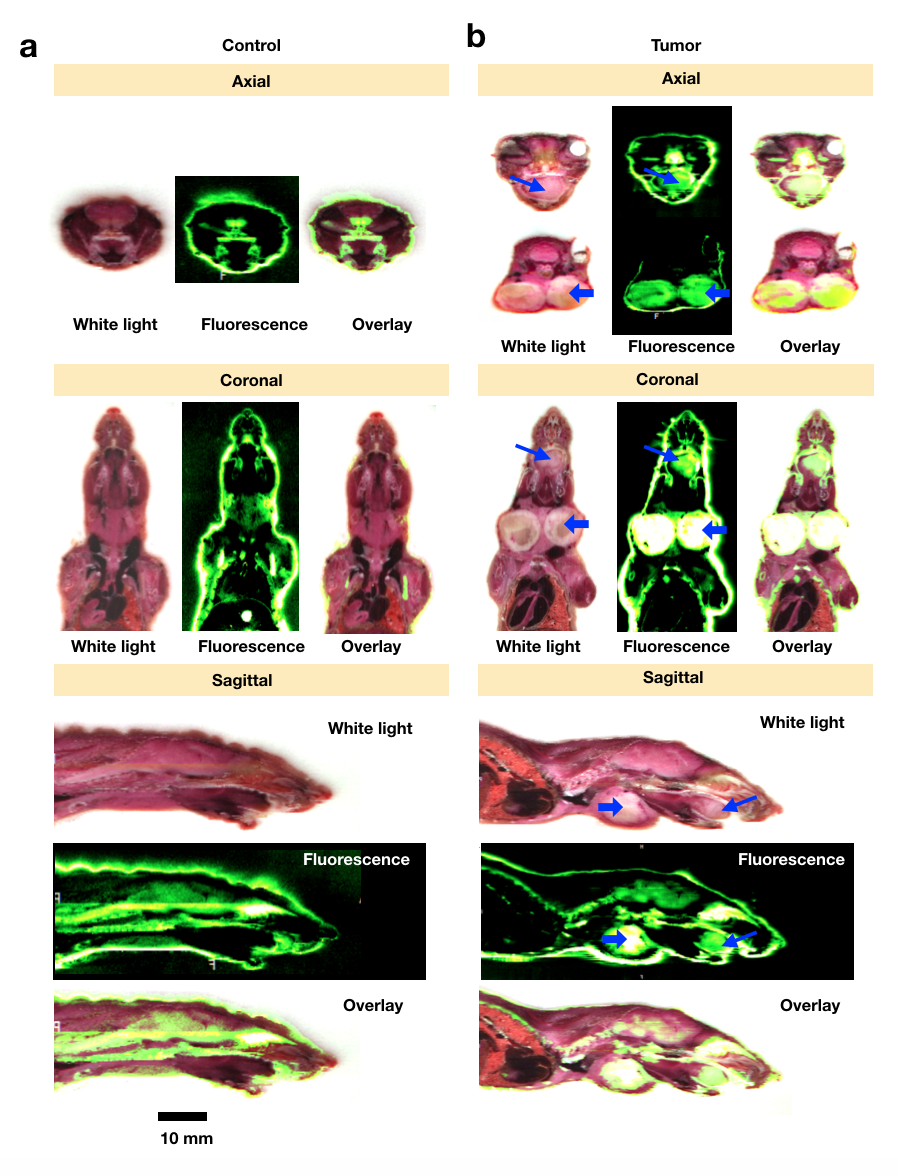
**

**Supplementary Fig 1.** Imaging of the head and neck region with PARPi-FL in a mouse with bilateral neck metastases. Axial (top), coronal (middle) and sagittal (bottom) views, 90 minutes post-intravenous-injection of 2.4 mg/kg of PARPi-FL (dissolved in 30% PEG in PBS). Mice were sacrificed 90 minutes post-injection, were flash-frozen at approximately -70 ˚C and processed for imaging. Images were acquired on a Xerra body sectioning and imaging system (EMIT imaging) using 470 nm excitation laser. **(a)** No accumulation of PARPi-FL was observed in a normal tongue muscle or in any other neck structures. Autofluorescence from the nasal turbinate, skin, whiskers, nose mucosa and from the tongue normal epithelium and gustative papillae were detected and also appear in green. **(b)** In a tumor-bearing mouse, both tumor and metastases presented with PARPi-FL uptake and emitted a green signal whereas the muscle and other structures did not. The narrow arrow points to tumor and the thicker arrow points to lymph node metastases. Just like for the control mouse, autofluorescence could be seen arising from nasal turbinate, skin, whiskers, nose mucosa and from the tongue normal epithelium and gustative papillae.


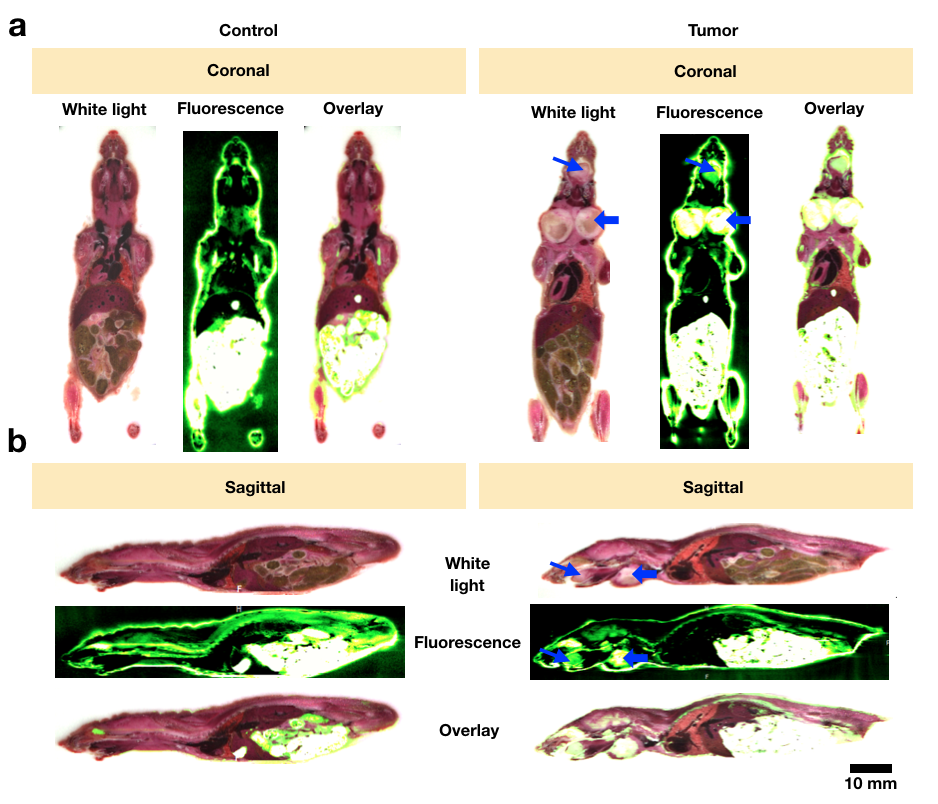


**Supplementary Fig 2.** Biodistribution of PARPi-FL in a mouse with bilateral neck metastases. Axial (top), coronal (middle) and sagittal (bottom) views, 90 minutes post-intravenous-injection of 2.4mg/kg of PARPi-FL (dissolved in 30% PEG in PBS). Mice were sacrificed 90 min post-PARPi-FL-injection, flash-frozen in hexanes at approximately -70 ˚C and processed for imaging. Images were acquired on a Xerra body sectioning and imaging system (EMIT imaging) using a 470 nm excitation laser. **(a)** No accumulation of PARPi-FL was observed in a normal tongue muscle or in any other neck structures. Autofluorescence from the nasal turbinate, skin, whiskers, nose mucosa and from the tongue normal epithelium and gustative papillae were detected and also appear in green. Significant autofluorescence is also seen in the small intestines, large intestines. PARPi-FL excretion was confirmed to be hepatobiliary – accumulation in the gallbladder. **(b)** In a tumor-bearing mouse, both tumor and metastases presented with PARPi-FL uptake and emitted signal in the green channel whereas the muscle and other structures did not. The narrow arrow points to tumor and the thicker arrow points to lymph node metastases. Just like for the control mouse, autofluorescence could be seen arising from nasal turbinate, skin, whiskers, nose mucosa and from the tongue normal epithelium, gustative papillae, small intestines, large intestines and gallbladder.


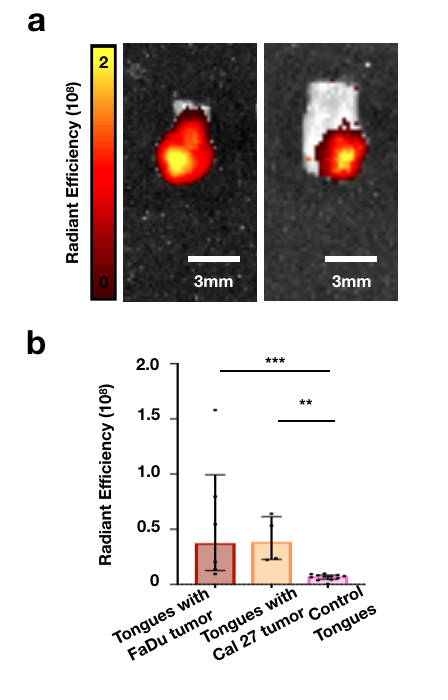


**Supplementary Fig. 3.** Tumor delineation with PARPi-FL. **(a)** PARPi-FL accumulated in the parts of the tongue where tumors were present, whereas no signal could be observed in areas with normal muscle or in the control tongues (larger tumor represented on the left and smaller tumor on the right). **(b)** The average epifluorescence in the tumor areas within the xenografted tongues was significantly higher (p < 0.001 for FaDu and p = 0.001 for Cal 27) than in the controls, yielding a tumor-to-background ratio of 7.64 (10.25 FaDu and 5.08 Cal 27). The tumor-to-background ratios allowed precise fluorescent delineation of the tumor within the tongues in all xenografted animals.


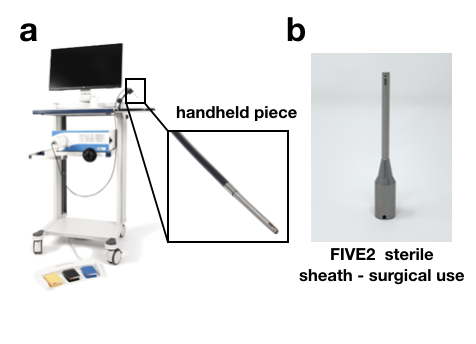


**Supplementary Fig. 4.** Hand-held confocal microscopy setup. **(a)** The portable small hand-piece of this confocal microscope potentially allows use in the operating room. Images are

generated by scanning with the fiberoptic tip and mapping a region of interest in real time. The equipment has a single channel for illumination and detection of PARPi-FL (488 nm excitation, 515 – 575 nm emission, 1 frame/s), and a NA of 0.3 (similar to a 10× objective) with a field of view of about 0.5 mm^2^. The probe needs to be in direct contact with the tissue to acquire images and contains a Z mechanism that can continuously scan at variable depths (Z axis, 0 µm - 400 µm). **(b)** The instrument sheath can be sterilized and attaches to the hand-held piece through a magnet. No loss in image quality was observed with the use of the sheath. A sterile sheath is fundamental to allow the device to be used during surgery.
